# Supervisor and coworker trust as predictors of work engagement and burnout among migrant workers: the moderating role of language proficiency and contact with co-national coworkers

**DOI:** 10.3389/fpubh.2026.1755139

**Published:** 2026-03-16

**Authors:** Michał Kulisz, Antoni Wontorczyk

**Affiliations:** 1 Doctoral School in Social Sciences, Jagiellonian University, Kraków, Poland; 2 Faculty of Management and Social Communication, Institute of Applied Psychology, Jagiellonian University, Kraków, Poland

**Keywords:** burnout, JD-R theory, language proficiency, migration, psychosocial resources, work engagement, workplace trust

## Abstract

**Introduction:**

Migration has become a crucial factor influencing social and economic landscapes in the Global North, creating challenges for workplace integration. In this study we describe how supervisor and coworker trust increase work engagement and decrease burnout among migrant workers.

**Methods:**

The hypotheses were tested with a cross-sectional study based on the Job Demands-Resources (JD-R) theory and using well established questionnaire tools.

**Results:**

We showed that secondary level factors: supervisor and coworker trust act either as resources (from β [standardized coefficient] = 0.126, CIs = [0.001, 0.326] to β = 0.302, CIs = [0.184, 0.421] for particular subscales) or demands (from β = −0.090, CIs = [−0.127, 0.025] to β = −0.270, CIs = [−0.298, −0.116] for particular subscales), depending on primary level variables, located in individual and environmental conditions. We further show how the primary level factors influence the aforementioned relationships: host-country language proficiency moderates the relationship between coworker trust and burnout (*B* [unstandardized measure] = 0.960; CIs = [0.002, 1.918]; *f*^2^ [standardized measure] = 0.242) and that contact with co-national coworkers (*B* = 0.315; CIs = [0.046, 0.584]; *f*^2^ = 0.215) moderates the relationship between coworker trust and burnout.

**Discussion:**

Results indicate that coworker trust positively predicts work engagement and reduces burnout, particularly for migrants with high host country's language proficiency, irrespective of work's dominant language proficiency. Conversely, limited interaction with co-national coworkers strengthen the effects of workplace distrust on burnout. Our findings suggest that fostering inclusive workplaces and linguistic support may increase migrants' psychosocial well-being. Supporting communication and in particular the development of trust in culturally diverse work environments can increase employee health and wellbeing by decreasing burnout (from 3% to 23% depending on its dimension) and enhancing engagement (from 12% to 23% depending on dimension). The results may also be an insight to organizational and public health policies creators, referring to United Nations' Sustainable 2 Development Goal 3: Good Health and Well-Being and 8: Decent Work and Economic Growth.

## 1 Introduction

International migration is a defining trend in contemporary societies and labor markets, particularly across the Global North. According to a report by the International Labour Organization (1), the worldwide stock of migrants in the labor force totaled 167.7 million in 2022, which is an increase by 11 million from 2017.

As a result, host countries face increasing challenges in promoting the health, inclusion, and wellbeing of migrant workers—a key dimension of public health under the United Nations Sustainable Development Goal 3 (SDG 3) (2). Among the barriers in workplace integration one can name heightened exposure to psychosocial stressors, such as nation-based discrimination (3), not receiving sufficient support (4, 5) or difficulties in communication due to cultural differences (6). Conversely, workplace relationships characterized by trust, respect, and inclusion can serve as vital psychosocial resources and are often seen as more significant for migrant employees than for native workers (7).

Such experiences stemming from the social environment can have an impact on migrants' health, wellbeing, attitudes, and behavior at work. In this article, we aim to focus on relationships of work engagement and burnout with particular social environmental variables. Moreover, we aim to identify some moderating factors which enable the use of resources or increase negative consequences of demands.

A well-established framework for studying work engagement and burnout, key variables in understanding workplace health and wellbeing, is the Job Demands-Resources theory developed by Bakker and Demerouti in cooperation with Schaufeli (8). The theory has been widely used in organizational psychology to describe the development process of these phenomena and to show their direct association with work performance and worker health (8–10). It posits that there are two crucial paths shaping employee wellbeing: a health impairment process and a motivational process (11–13). In the first path, *resources* available to the individual lead to more engagement and buffer negative consequences of workplace demands. In the second path, *demands* increase the level of burnout and have an opposite effect on work engagement.

Work engagement is defined by Schaufeli et al. (14) as a “positive, fulfilling, work-related state of mind that is characterized by vigor, dedication, and absorption.” These three dimensions of work engagement are based upon three processes: emotionally-motivational, cognitive, and physiological-behavioral (15). Among the benefits of work engagement one can mention fewer sick leaves and better subjective health (9), as well as organizational outcomes like increased job performance or organizational commitment (16).

Burnout on the other hand, is defined by Schaufeli et al. (17) as a “work-related state of exhaustion that occurs among employees, which is characterized by extreme tiredness, reduced ability to regulate cognitive and emotional processes, and mental distancing.” Besides negative impacts on wellbeing and health such as insomnia and depressive states (8), consequences of burnout are also visible in the workplace with, e.g., higher absenteeism and decreased job performance (18).

Among many workplace environment resources trust in both supervisors and coworkers represents a crucial psychosocial factor in influencing engagement and burnout, particularly for migrant employees in culturally and linguistically diverse environments. Previous research has shown that both kinds of relationships—with coworkers and supervisors should be treated as separate constructs (19). Interactions with one's supervisor, often operationalized as leader-member exchange (LMX) (20) are more vertical and often bring opportunities, information, and support to subordinates in exchange for their proactive behavior and commitment (21). On the other hand, relationships with coworker's team-member exchange (TMX) are usually more horizontal and equal, providing more informal and emotional kinds of support (19). Furthermore, a meta-analysis by Banks et al. (19) indicates that LMX and TMX are distinct constructs. LMX has usually a bigger impact on performance and turnover, while TMX provides incremental prediction for commitment and satisfaction in some cases.

Migrant workers' trust in their supervisors can develop at various levels of relationship. Typically, workplace interactions remain limited to common supervisory tasks, such as assisting with work-related tasks. In some cases, supervisors also provide additional support, e.g., language translations or clarifying informal workplace rules and practices. Some scholars, however, describe forming a more personal relationship between migrants and their employers, where the latter guide migrants on their host country's culture adjustment, support their professional growth, and even offer help with personal matters outside of work (22, 23). Conversely, negative experiences such as nationality-based discrimination (3) or marginalization (4, 5) have also been documented. Lack of trust can have significant widespread and lasting consequences at the workplace. Bazzoli and Probst (24) identify distrust as a crucial barrier inhibiting communication between management and migrant employees. Moreover, Li and Frenkel (25) report that many migrants struggle to build trust-based relationships with coworkers and managers, which often leads to social exclusion. On the other hand, perceived supervisor support and trust can promote migrants' cultural competence (26), a valuable resource in improving works effectiveness and engagement (27, 28). Additionally, Leader-Member Exchange, a broader concept that includes mutual trust, has been shown to positively influence migrant workers' wellbeing (26).

Relationships between coworkers as those having “no formal authority over one another” (29) can be developed on many layers as well. They can range from interactions solely related to conducting work, like passing information, support in work-tasks and giving feedback, to bond-forming activities, like self-disclosure or support on family issues (30). On the other hand, negative phenomena, like discrimination and stereotyping, misunderstandings, conflicts and group categorizations (3, 6) can also erode trust and have a profound impact on the intercultural workplace relations.

Lack of trust between native and migrant coworkers can lead, as mentioned earlier, to social exclusion of the latter (25). Furthermore, as Lahti and Valo (6) show, cultural differences can cause misunderstandings regarding how trust should be formed between colleagues of different nations. These, in turn can influence forming unfavorable opinions about coworkers, evoke negative stereotypes and even cause workplace conflicts. On the other hand, research on the general population has indicated that high coworker trust can be associated with more organizational citizenship behaviors (31) and fosters cooperative behavior (32).

As supervisor and coworker trust can have various benefits when they reach high value or negative consequences when they are low. We interpret them as resources (trust) or demands (distrust) in the understanding of the JD-R theory (8) dependent on their value. Given the above, we hypothesize that:

H1: *Supervisor trust and* c*oworker trust will positively predict work engagement and negatively predict burnout among migrant employees*.

The relationship between trust and engagement/burnout can also be dependent on other factors stemming from the individual. Language proficiency represents an additional individual resource that may shape how workers utilize social resources at work. As shown in numerous studies, such proficiency can be a significant resource when working in the receiving country. It may not only enhance the use of workplace equipment and facilities (33) but also facilitates effective communication (34). Particularly, language proficiency can significantly impact the quality of relationships (6) and support received from one's work colleagues (35), as well as increase the value of work-resources gained from interacting with one's supervisor (36). One may notice that language proficiency is not necessary to conduct many work duties, especially in blue-collar jobs. As Lahti and Valo (6) report, coworkers who are not able to communicate verbally, use non-verbal communication not only for professional matters but also to convey emotions, respect or support. However, such methods are not an effective substitute of verbal communication when it comes to workplace-relationship building or communicating complex ideas. Those studied workers who were unable to communicate with their colleagues admitted that they avoided more complex and unnecessary interaction due to stress and embarrassment which they have experienced during unsuccessful conversation attempts (6). Moreover, as Faaliyat et al. (37) argue, lack of language proficiency also limits the worker's involvement in decision-making.

As mentioned above, language proficiency is not necessary for conducting many kinds of work, though its use may strengthen the ability to use workplace interactions as a resource. In other words, it does not create trust or engagement by itself but rather creates conditions which allow using trust as a resource in enhancing work engagement. We therefore hypothesize as follows:

H2a: *Proficiency in the host country's language will moderate the relationship between coworker and supervisor trust and work engagement. The relationship will be stronger for workers who are proficient in using the language*.

In multilingual and globalized work environments, the primary workplace language may differ from the host-country language. To enable more effective communication, multinational companies serving international markets often decide to use English or the main language of the client for communication, both for external and internal conversations (38). In consequence, the host country's language may lose its relevance. We therefore also hypothesize that:

H2b: *Proficiency in the workplace's dominant language will moderate the relationship between coworker and supervisor trust and work engagement. The relationship will be stronger for workers who are proficient in using the language*.

Finally, the social structure of the workplace may also influence how migrants experience trust, which in consequence influences their engagement or burnout. Nationally and culturally diverse workplaces offer many opportunities for intercultural communication, however one can name various reasons why a migrant worker might prefer relationships with their co-national coworkers over interacting with native colleagues. Since migrants often find work thanks to their migration networks—their friends and relatives who already have experience with working in the host country, some acquaintances can be made prior to arriving and then prevail in the workplace (39). Another reason for choosing primarily fellow nationals for workplace interactions may be language and cultural differences. As it has been described earlier, these are often perceived as obstacles due to complicating communication (6).

In consequence, those who have the opportunity to interact with fellow nationals in their workplace may choose these interactions over relationships with colleagues of other nationalities, resembling the *separation* strategy described in Berry's acculturation model (40). This in turn may lower the perceived significance of trust in coworkers and workplace social climate as one can have a minority-group of favored colleagues and to some extent ignore the rest of work's social environment. Such a pattern has indeed been identified in in-depth interviews with temporary migrant workers by Lahti and Valo (6), who described such behavior as forming *cultural cliques*. The interviewed workers have based most “comfort, assistance and self-validation” (6) on their national minority, omitting interactions with natives from the host country. On the other hand, among those who do not interact with fellow national coworkers, distrust may bear more emotional costs due to lack of identity-based support and stress buffering, especially among those who do not integrate with the host country's society and adopt a separation (40) strategy.

Rather than influencing trust directly, contact with co-national coworkers may therefore create conditions in which trust in coworkers might have less meaning, as it would have without such contact. We thus hypothesize that:

H3: *Contact frequency with co-national coworkers will moderate the relationship between coworker trust, workplace climate, and burnout. The relationship will be stronger among those who do not interact with their co-national colleagues*.

## 2 Materials and methods

### 2.1 Study objective

This study aims to assess how supervisor and coworker trust relate to work engagement and burnout among migrants, and to test whether proficiency in the language of the receiving country (host country's language proficiency) and the language at one's workplace (work language proficiency), as well as contact with co-national coworkers, moderate these relationships.

### 2.2 Participant recruitment

A cross-sectional online survey was conducted as part of a broader research project between June 2024 and August 2024 among Polish migrant workers living in 27 host countries. Recruitment occurred via social media groups dedicated to the Polish diaspora and through snowball sampling. Prior to participation, individuals were informed about the eligibility criteria, which included employment abroad for at least 2 months and age 18 or above. All participants provided written informed consent before taking part in the research and shared the data voluntarily, assured that their data would only be published in an anonymized form.

### 2.3 Ethical approval

The study received approval by the ethical committee of the Faculty of Management and Social Communication at the Jagiellonian University in Kraków, Poland on 01.03.2024 (decision no. 19/2024). All procedures followed relevant ethical guidelines and regulations.

### 2.4 Participants

**Figure 1:**
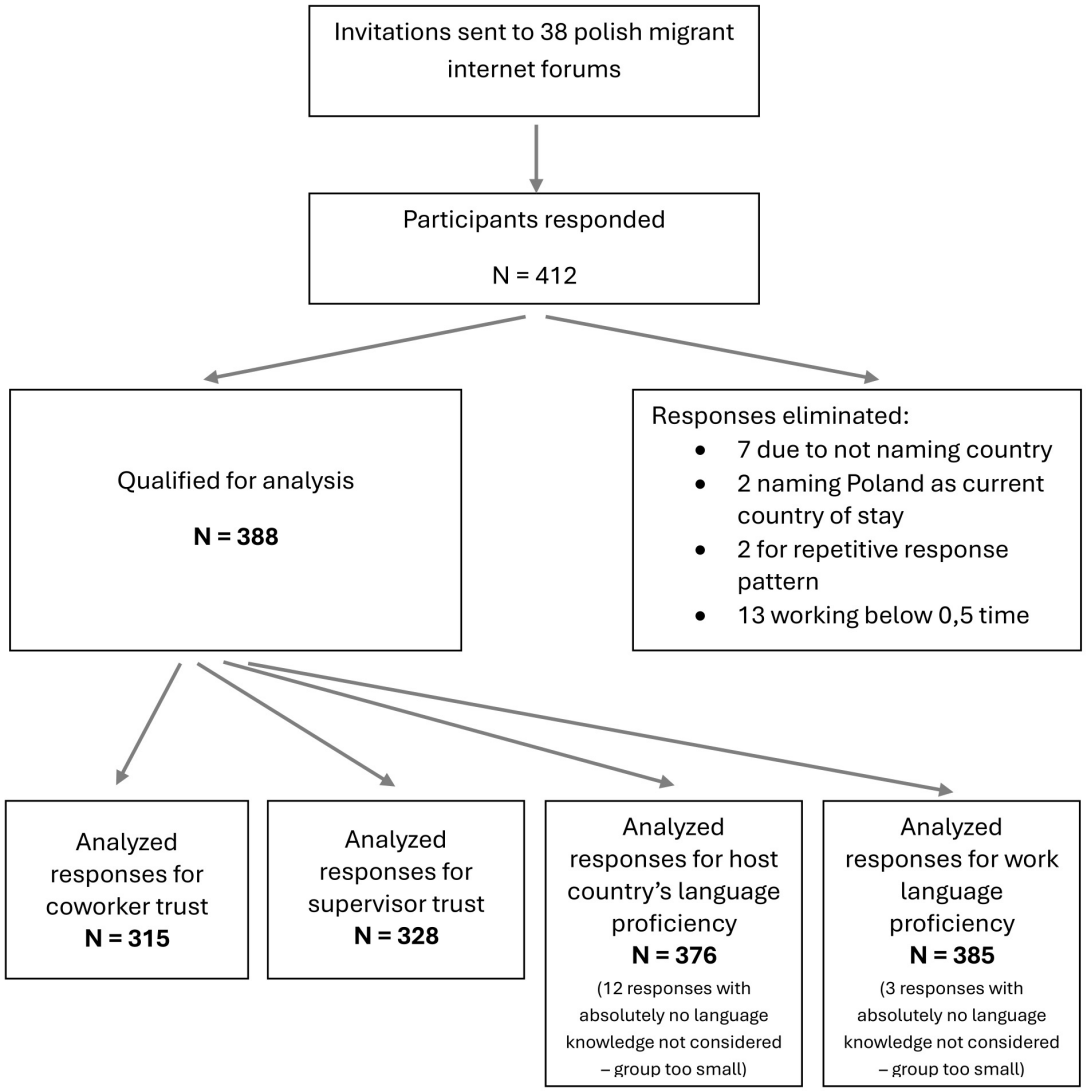
Study recruitment flow chart.

Data from 412 respondents were collected. Responses from 7 participants were excluded due to not reporting country of stay and 2 were excluded due to naming Poland as their country of stay. Thirteen participants who indicated that they worked below 0.5 time and 2 participants who showed invariant response patterns were excluded as well. A detailed recruitment flow diagram is presented in Figure 1. The final sample consisted of 388 migrants, including 103 men and 284 women, with one participant not disclosing gender. The mean age was 40.41 years (SD = 11.02), while the average length of stay abroad was 11.30 years (SD = 1.48) and 10.45 years (SD = 1.24) in the current host country. 38.4% participants indicated that they had no contact with co-national coworkers, 31.2% claimed to interact with other Polish coworkers sporadically, 30.4% reported frequent contact. 82.0% respondents claimed to have a high host country's language proficiency, 14.9% claimed to know and use single words or have a low language proficiency, finally 3.1% claimed absolutely no host country's language knowledge. 74.2% study participants reported proficient knowledge of work language, 25.0% limited and 0.7% none. As only a very small group of participants indicated absolutely no language knowledge, we decided not to include this group in analyses regarding language skills. Furthermore, respondents reported their employment statuses as follows: 71.6% were full-time workers, 9.3% part-time workers, and 19.1% participants with undefined schedules. Regarding education, 67.6% had higher education, 22.1% completed secondary education, 6.3% had technical or industry-specific secondary education, 2.7% reported vocational education, and 1.3% other types of education. Most participants (84.4%) worked for private companies, while 15.6% were employed in public or state-owned organizations. Respondents were located across 27 countries, with Germany (14.1%), Iceland (11.9%), Italy (11.4%), Greece (7.8%), and the United Kingdom (7.0%) being the most commonly mentioned. Additional locations included North America (e.g., 5.8% from both USA and Canada), Asia (e.g., 2.9% in Singapore), and Australia (6.8%). Other locations were named by 26.5% respondents.

### 2.5 Measures

Four validated tools were used for data collection: a socio-demographic questionnaire, a work engagement survey, a burnout scale, and the second edition of the Copenhagen Psychosocial Questionnaire (COPSOQ-II). Norms for interpretation of results can be found in Table 1. The norms were provided by authors of the questionnaires in the user manuals which were mentioned in the tool descriptions of this article. The norms are based on samples from the validation studies of the tools. If a result is equal to the results obtained lower quartile of the validation sample, it indicates a low score, whereas reaching results equal to upper quartile indicates high score.

**Table 1:** Norms for interpretation of study variables.

**Variable**	***M***	Score norms				
		**Min score**	**Low**	**Medium**	**High**	**Max score**
Work engagement general scale	30.33	0	≤15.93	26.01–41.94	≥42.09	45
Vigor	10.20	0	≤9.75	9.78–11.40	≥14.43	15
Dedication	10.67	0	≤9.70	8.73–14.10	≥14.13	15
Absorption	9.46	0	≤6.99	7.02–12.60	≥12.23	15
Burnout general scale	24.33	11	≤22.80	22.92–33.24	≥33.36	55
Coworker trust	6.51	3	≥9.00	8.88–6.00	≤5.88	15
Supervisor trust	9.19	3	≥11.36	10.88–7.04	≤6.88	15

The interpretation norms were provided by authors of the questionnaires in the user manuals which were mentioned in the tool descriptions of this article. The norms are based on samples from the validation studies of the tools. If a result is equal to the results obtained lower quartile of the validation sample, it indicates a low score, whereas reaching results equal to upper quartile indicates high score. For the language version of BAT-C used in the study, authors of the tool provided only norms for the burnout general scale.

The minimum and maximum scores are both: scores possible to achieve and scores obtained by study participants.

The results of supervisor trust and coworker trust have not been reversed for analysis purposes, therefore the lower the score on those scales, the better the level of trust. For further analyses (correlation, regression, moderation) the scales have been reversed.

#### 2.5.1 Co-national coworker contact and language proficiency

A socio-demographic form developed by the authors captured key variables, including Polish co-national coworker contact frequency and work and host country's language proficiency. The respondents were asked to choose respectively whether they interact with Polish coworkers often, sporadically, or never. They also assessed their knowledge of the primary work language and host country's language in the same manner: as either proficient or sufficient for basic communication/limited to a few words. We chose this brief method of language proficiency assessment to minimize attrition rates. We also did not choose to ask participants to provide any language certificates, as this would have limited the respondents group to those who had a motivation to prove their language proficiency.

#### 2.5.2 Work engagement

Work engagement was evaluated using the Polish version of the Utrecht Work Engagement Scale (UWES-9), adapted by Szabowska-Walaszczyk et al. (41). This 9-item survey, a shortened form of the UWES measure (42), required participants to rate statements on a 7-point Likert scale ranging from 0 (“never”) to 6 (“always/every day”). The scale measures three dimensions of work engagement—*Vigor, Dedication*, and *Absorption*—with each dimension assessed by three items. The overall reliability of the scale was high (Cronbach′s α = 0.92).

#### 2.5.3 Burnout

Burnout was assessed using the Polish version of the Burnout Assessment Tool (BAT) (43), originally developed by Schaufeli et al. (44), with the study employing the shortened BAT-C version. We decided to use the Tool due to its alignment with the JD-R theory (45), as well as highly satisfying psychometric properties (43, 44). The shortened version evaluates core burnout symptoms across four subscales: Exhaustion, Mental Distancing, Cognitive Impairment, and Emotional Impairment, with three items per subscale. Responses were recorded on a 5-point Likert scale ranging from 1 (“never”) to 5 (“always”). Reliability scores were Cronbach′s α = 0.95 for the overall scale and ranged from 0.83 to 0.92 for individual subscales.

#### 2.5.4 Coworker and supervisor trust

Coworker trust and supervisor trust were measured using the *Mutual trust between employees* and *Vertical trust* scales from the Polish version of COPSOQ-II (46), originally developed by Pejtersen et al. (47). Each scale contained three items, with responses on a 5-point Likert scale ranging from 1 (“always”) to 5 (“never/almost never”). As the study was aimed at including migrants working single-handedly (e.g., single entrepreneurs), the scales were optional to answer. Cronbach's α for the Vertical trust was 0.74 and for Mutual trust between employees −0.68 (both for Polish versions). Although for Mutual trust Cronbach's α was slightly below conventional thresholds, McDonald's ω indicated adequate composite reliability (ω = 0.77). In our study results, Cronbach's α reached 0.84 for both scales.

### 2.6 Statistical analysis

Pearson's correlations, multiple regression, and moderation analyses were conducted separately for general scores of work engagement and burnout, as well as all of their subscales, using SPSS and Hayes' PROCESS macro. Significance was determined at α = 0.05. Interactions were probed using conditional effects for moderator levels.

Among all study variables, only trust scales could be filled voluntarily. As the participants could only submit their questionnaires when all scales (besides trust) were answered, there is no missing data to be reported. Analyses for trust scales were conducted using listwise deletion of observations with missing trust data.

It should be noted that within the presentation of descriptive statistics (Tables 1, 2) the results for supervisor and coworker trust have not been reversed, therefore the lower the score on those scales, the more positive the (coworker and supervisor) trust. For further analyses of correlation, regression, and moderation (Tables 3–6) the results have been reversed, in consequence the lower the score on those scales, the more negative the (coworker and supervisor) trust.

**Table 2:** Descriptive statistics of the studied variables.

**Variable**	***N***	***M***	**SD**	95% CI of M		**SE**	**Min**.	**Max**	**Skewness**	**Kurtosis**	***D***	***p***
				LL	UL							
Work engagement general scale	388	30.33	9.91	28.72	30.89	9.902	0	45	−0.707	0.070	0.095	0.001
Vigor	388	10.20	3.57	9.56	10.35	3.568	0	15	−0.738	0.187	0.121	<0.001
Dedication	388	10.67	3.65	10.13	10.92	3.652	0	15	−0.773	−0.089	0.136	<0.001
Absorption	388	9.46	3.83	8.90	9.75	3.828	0	15	−0.622	−0.152	0.106	<0.001
Burnout general scale	388	24.33	6.46	23.98	25.41	6.463	11	55	0.481	0.724	0.057	0.138
Exhaustion	388	7.95	2.72	7.84	8.45	2.718	3	15	0.424	−0.245	0.126	<0.001
Distancing	388	5.33	1.88	5.14	5.55	1.881	3	15	0.125	−0.461	0.112	<0.001
Cognitive impairment	388	5.96	2.04	5.77	6.22	2.037	3	15	0.558	0.659	0.150	<0.001
Emotional impairment	388	5.09	2.01	4.98	5.43	2.011	3	15	0.992	0.958	0.161	<0.001
Coworker trust	315	6.51	3.01	6.18	6.84	3.008	3	15	0.931	0.154	0.186	<0.001
Supervisor trust	328	9.19	3.61	8.85	9.64	3.606	4	20	0.671	0.118	0.099	<0.001

D and p according to the Kolmogorov-Smirnov test.

The number of respondents in the analysis of supervisor trust and coworker trust is lower since the scales were not obligatory to fill out for people working solitarily.

The results of supervisor trust and coworker trust have not been reversed for analysis purposes, therefore the lower the score on those scales, the better the level of trust. For further analyses (correlation, regression, moderation) the scales have been reversed.

**Table 3:** Correlations between the studied variables.

**Independent variables**	**Dependent variables**								
	**WE general scale**	**WE Vigor**	**WE Ded**.	**WE Abs**.	**B general scale**	**B Exh**	**B Dist**	**B Cogn. imp**.	**B Emot. imp**.
Coworker trust	0.402^**^	0.408^**^	0.403^**^	0.272^**^	−0.405^**^	−0.422^**^	−0.347^**^	−0.154^*^	−0.240^**^
Supervisor trust	0.439^**^	0.434^**^	0.421^**^	0.327^**^	−0.388^**^	−0.418^**^	−0.300^**^	−0.143^*^	−0.242^**^

For coworker trust N = 315 (df = 313), for supervisor trust N = 328 (df = 326), the scales were not obligatory for those working solitarily.

^*^significant at 0.01, ^**^significant at 0.001.

## 3 Results

### 3.1 Preliminary analysis

Table 2 displays descriptive statistics of the main study variables. To ensure data presentation consistency, all presented mean scores were obtained by calculating mean scores of summed scores from all items of a scale. The respondents demonstrated on average a moderate level of work engagement [*M* = 30.33; CIs = (28.72, 30.89); SD = 9.91] and burnout [*M* = 24.33; CIs = (23.98, 25.41); SD = 6.46], as well as of supervisor [*M* = 9.19; CIs = (8.85, 9.64); SD = 3.61] and coworker trust [*M* = 6.51; CIs = (6.18, 6.84); SD = 3.01]. Detailed norms for results interpretation can be found in Table 1.

Intercorrelations between the studied continuous variables are presented in Table 3. All correlations were significant and almost all had a moderate or a low level.

To check the prediction between the studied variables a multiple regression analysis using enter selection method has been conducted. To check the assumptions for regression analysis, we conducted an optical analysis of homoskedasticity and residual plots prior to running the analysis. The results of the analysis are presented in Table 4.

**Table 4:** Regression effects between the studied variables.

**Dependent variable**	**Ind. variable**	**β**	***B***	**B outside of model**	***t***	95% CI		***p***	**adj. R^2^**	**ΔR^2^**	**F of Δ**	**F of Δ signif**.	***f^2^***	**Power (1- β)**
						**LL**	**UL**							
Burnout: exhaustion	Coworker trust	−0.263	−0.242	−0.387	−4.36	−0.351	−0.133	<0.001	0.226	0.226	47.51	<0.001	0.292	1.00
	Supervisor trust	−0.270	−0.207	−0.319	−4.49	−0.298	−0.116	<0.001						
Burnout: distancing	Coworker trust	−0.257	−0.163	−0.221	−4.05	−0.243	−0.084	<0.001	0.135	0.140	26.61	<0.001	0.156	1.00
	Supervisor trust	−0.160	−0.085	−0.157	−2.53	−0.151	−0.019	0.012						
Burnout: cognitive impairment	Coworker trust	−0.101	−0.069	−0.105	−1.50	−0.160	0.022	0.135	0.023	0.029	4.86	0.008	0.024	0.68
	Supervisor trust	−0.090	−0.051	−0.081	−1.33	−0.127	0.025	0.185						
Burnout: emotional impairment	Coworker trust	−0.135	−0.094	−0.166	−2.06	−0.184	−0.004	0.041	0.074	0.074	13.01	<0.001	0.080	1.00
	Supervisor trust	−0.169	−0.098	−0.139	−2.57	−0.173	−0.023	0.011						
Work eng: vigor	Coworker trust	0.233	0.281	0.490	3.88	0.138	0.423	<0.001	0.224	0.228	48.27	<0.001	0.289	1.00
	Supervisor trust	0.302	0.302	0.433	5.02	0.184	0.421	<0.001						
Work eng: dedication	Coworker trust	0.235	0.282	0.483	3.88	0.139	0.425	<0.001	0.215	0.220	45.93	<0.001	0.274	1.00
	Supervisor trust	0.291	0.291	0.420	4.81	0.175	0.410	<0.001						
Work eng: absorption	Coworker trust	0.126	0.162	0.350	1.96	0.001	0.326	0.051	0.111	0.116	21.39	<0.001	0.125	1.00
	Supervisor trust	0.251	0.270	0.351	3.90	0.134	0.406	<0.001						

VIF for predictors = 1.528.

The number of respondents in the analysis (*N* = 321; df1 = 2, df2 = 318) is lower since the scales were not obligatory to fill out for people working solitarily. Only those who filled all scales were considered for analysis.

Supervisor trust entered all regression equations and was the stronger predictor than coworker trust for most subscales of burnout and engagement, reaching from small effects like β = −0.090 [CIs = (−0.127, 0.025)] for burnout's Cognitive impairment subscale to medium-sized like β = 0.302 [CIs = (0.184, 0.421)] for Vigor (work engagement subscale). Coworker trust predicted all subscales of burnout and engagement besides the Absorption subscale, where it was on the verge of prediction significance.

We have additionally conducted an attrition analysis, examining the differences between those who decided to report their trust among coworkers and trust for supervisors and those who did not share their answer. The results indicate that those who did not report their trust among coworkers were less engaged [M difference for engagement general scale = 2.70, *t*_(386)_ = 2.16, *p* = 0.016] and more burned out [M difference for burnout general scale = 1.93, *t*_(386)_ = −2.37, *p* = 0.009]. Similarly, those who did not report their trust for their supervisor were more burned out [M difference for burnout general scale = 2.64, *t*_(386)_ = −3.06, *p* = 0.001]. No significant difference in age was observed, no remarkable differences could be spotted in gender and education as well.

### 3.2 Moderation analysis

**Figure 2:**
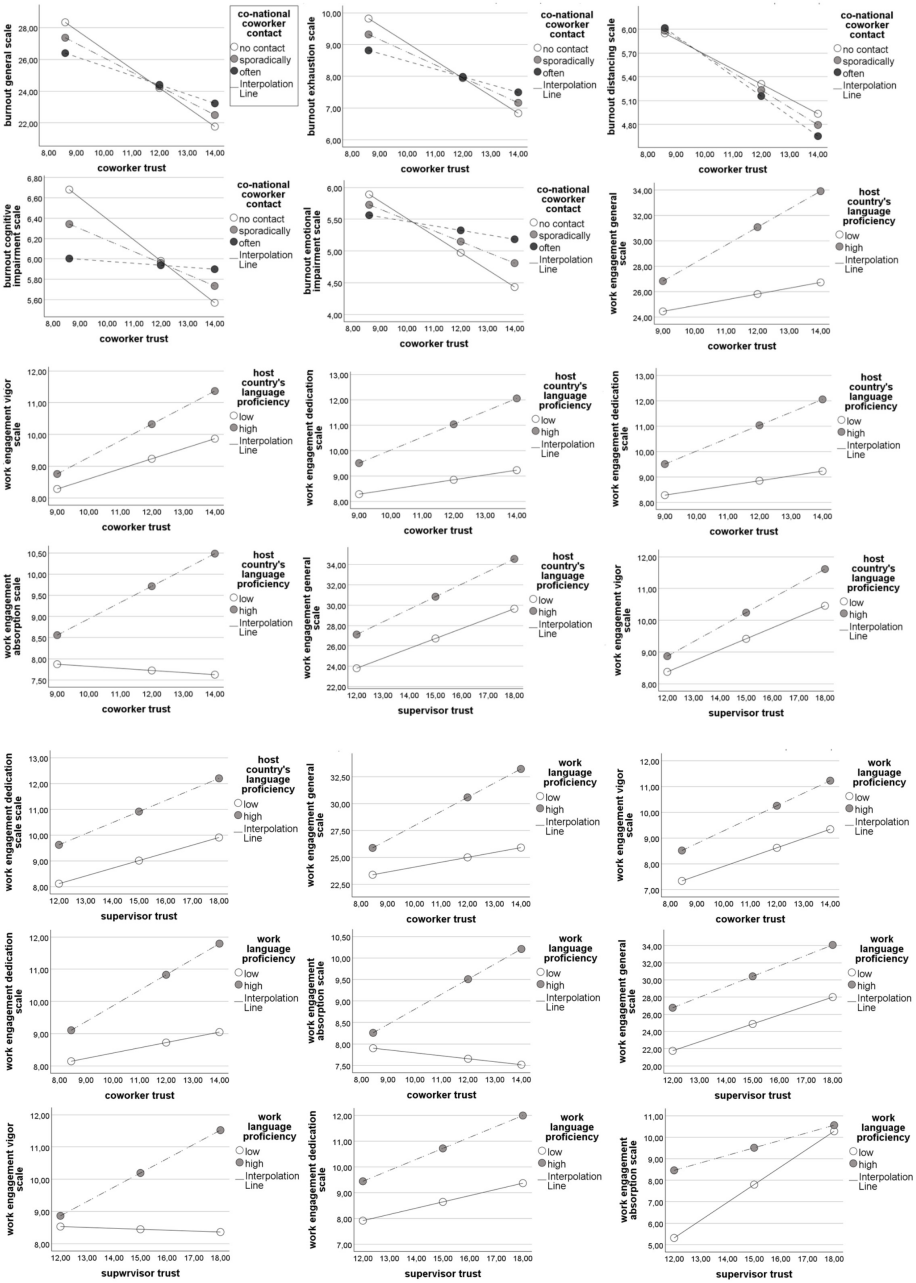
Slope plots of the studied moderations.

A series of moderation analyses were conducted to examine the moderating effects of co-national coworker contact and (work and host country's) language proficiency on coworker and supervisor trust, and both burnout and work engagement. Below we report results for each model, which have also been presented in Table 5. Results of conditional effects at different values of the moderator for significant interactions can be found in Table 6. Interaction plots presenting the studied moderations can be found in Figure 2. Unstandardized coefficients (B) are reported to facilitate interpretation of effects, while Cohen's f^2^ is reported to quantify the overall effect size of the regression models.

**Table 5:** Moderation effects between the studied variables.

**Moderator**	**Ind. variable**	**Dep. variable**	**Df**	***B***	**SE**	***t***	***p***	95% CI		**ΔR^2^**	**F of Δ**	**p of Δ**	***f^2^***	**Power (1- β)**
								**LL**	**UL**					
Co- national coworker contact	Coworker trust	Burnout general scale	2.312	0.315	0.137	2.30	0.022	0.046	0.584	0.013	5.31	.022	0.215	1.00
		Exhaustion	2.312	0.154	0.057	2.70	0.007	0.042	0.266	0.018	7.27	.007	0.243	1.00
		Distancing	2.312	−0.032	0.041	−0.78	0.434	−0.114	0.049	0.002	0.61	.434	0.140	1.00
		Cognitive impairment	2.312	0.093	0.047	2.01	0.045	0.002	0.185	0.012	4.04	.045	0.037	0.87
		Emotional impairment	2.312	0.100	0.046	2.15	0.032	0.009	0.191	0.013	4.64	0.032	0.069	0.99
Host country's language proficiency	Coworker trust	Work engag. general scale	2.307	0.960	0.487	1.97	0.050	0.002	1.918	0.010	3.88	0.050	0.242	1.00
		Vigor	2.307	0.206	0.178	1.15	0.249	−0.145	0.556	0.003	1.33	0.249	0.229	1.00
		Dedication	2.307	0.320	0.178	1.80	0.073	−0.030	0.670	0.008	3.23	0.073	0.249	1.00
		Absorption	2.307	0.434	0.199	2.18	0.030	0.042	0.826	0.013	4.75	0.030	0.116	1.00
	Supervisor trust	Work engag. general scale	2.314	0.264	0.375	0.70	0.483	−0.474	1.001	0.001	0.49	0.483	0.296	1.00
		Vigor	2.314	0.110	0.137	0.80	0.424	−0.160	0.380	0.002	0.64	0.424	0.272	1.00
		Dedication	2.314	0.131	0.138	0.95	0.343	−0.141	0.404	0.002	0.90	0.343	0.277	1.00
		Absorption	2.314	0.022	0.154	0.14	0.885	−0.281	0.325	0.000	0.02	0.885	0.155	1.00
Work language proficiency	Coworker trust	Work engag. general scale	2.310	0.867	1.45	0.60	0.550	−1,984	3.718	0.001	0.36	0.550	0.200	1.00
		Vigor	2.310	0.127	0.528	0.24	0.810	−0.912	1.165	0.000	0.06	0.810	0.193	1.00
		Dedication	2.310	0.321	0.528	0.61	0.544	−0.718	1.359	0.001	0.37	0.544	0.202	1.00
		Absorption	2.310	0.420	0.595	0.71	0.481	−0.751	1.591	0.001	0.50	0.481	0.084	1.00
	Supervisor trust	Work engag. general scale	2.324	0.177	1.25	0.14	0.888	−2.284	2.637	0.000	0.02	0.888	0.255	1.00
		Vigor	2.324	0.471	0.456	1.03	0.302	−0.426	1.369	0.003	1.07	0.302	0.250	1.00
		Dedication	2.324	0.183	0.460	0.40	0.692	−0.723	1.088	0.000	0.16	0.692	0.230	1.00
		Absorption	2.324	−0.477	0.514	−0.93	0.354	−1.489	0.534	0.002	0.86	0.354	0.132	1.00

The number of respondents in the analysis is different between the scales coworker trust and supervisor trust since the scales were not obligatory to fill out for people working solitarily. Moreover, the number of respondents is lower for the analyses of both types of language proficiency, since few respondents indicated no language knowledge. Only those who filled both scales (independent variable and moderator) scales were considered for analyses.

**Table 6:** Conditional effects at different values of the moderator for significant interactions.

**Moderator**	**Ind. variable**	**Dependent variable**	**Moderator value**	***B***	**SE**	***t***	***p***	**95% LLCI**	**95% ULCI**
Co-national coworker contact	Coworker trust	Burnout general scale	No contact	−1.216	0.182	−6.68	<0.001	−1.574	−0.858
			Sporadically	−0.901	0.109	−8.25	<0.001	−1.116	−0.686
			Often	−0.586	0.168	−3.50	<0.001	−0.916	−0.257
		Burnout: exhaustion	No contact	−0.552	0.076	−7.26	<0.001	−0.702	−0.403
			Sporadically	−0.398	0.046	−8.73	<0.001	−0.488	−0.309
			Often	−0.245	0.070	−3.49	<0.001	−0.382	−0.107
		Burnout: cognitive impairment	No contact	−0.206	0.062	−3.33	0.001	−0.328	−0.085
			Sporadically	−0.113	0.037	−3.04	0.003	−0.186	−0.040
			Often	−0.019	0.057	−0.34	0.733	−0.132	0.093
		Burnout: emotional impairment	No contact	−0.270	0.062	−4.37	<0.001	−0.392	−0.148
			Sporadically	−0.170	0.037	−4.59	<0.001	−0.243	−0.097
			Often	−0.070	0.057	−1.23	0.218	−0.182	0.042
Host country's language proficiency	Coworker trust	Work engagement general scale	Low	0.457	0.453	1.01	0.314	−0.435	1.349
			High	1.417	0.178	7.96	<0.001	1.066	1.767
		Work engagement: absorption	Low	−0.049	0.186	−0.27	0.791	−0.414	0.316
			High	0.385	0.073	5.29	<0.001	0.242	0.528

#### 3.2.1 Host country's language proficiency as a moderator

The moderation of the relationship between coworker trust and the general scale of work engagement by host country's language proficiency was found to be significant. The overall model demonstrated a moderate effect size [*f*^2^ = 0.242, *B* = 0.960 SE = 0.487, *t* = 1.97, CIs = (0.002, 1.918)]. Conditional effects showed that coworker trust's positive association with engagement was significant and stronger for those with high language proficiency [*B* = 1.417, SE = 0.178, *t* = 7.96, CIs = (1.066, 1.767)], contrary to those with low proficiency, for whom values were low and not significant [*B* = 0.457, SE = 0.453, *t* = 1.01 CIs = (−0.435, 1.349)]. Significant moderation effects with low-sized model effects size have also been found for the Absorption subscale [*f*^2^ = 0.116, *B* = 0.434, SE = 0.199, *t* = 2.18, CIs = (0.042, 0.826)], which followed the same pattern of conditional effects.

No significant moderation interactions have been found for host country's language proficiency moderating the relationship between supervisor trust and work engagement. Similarly, no significant moderation interactions have been found for work language proficiency moderating the relationship between both coworker and supervisor trust and work engagement. We also decided to conduct a brief *post-hoc* analysis using *t*-tests to check if the two work language proficiency groups (proficient/not proficient) differ in the value of the study variables. For host country's language proficiency, no significant difference could be observed in work engagement (*t* = 1.819, *p* = 0.070), burnout (*t* = 0.011, *p* = 0.991), as well as coworker (*t* = 0.821, *p* = 0.412) and supervisor trust (*t* = 0.247, *p* = 0.805).

#### 3.2.2 Co-national coworker contact as moderator

The relationship between coworker trust and the general burnout scale was significantly moderated by co-national coworker contact in a model reaching a medium-sized effect [*f*^2^ = 0.215, *B* = 0.315, SE = 0.137, *t* = −2.30, CIs = (0.046, 0.584)]. Conditional effects indicate that coworker trust was most strongly, negatively associated with burnout when contact was absent [*B* = −1.216, SE = 0.182, *t* = 6.68, CIs = (−1.574, −0.858)], with diminishing effects for sporadic [*B* = −0.901, SE = 0.109, *t* = 8.25, CIs = (−1.116, −0.686)] and frequent contact [*B* = −0.586, SE = 0.168, *t* = 3.50, CIs = (−0.916, −0.257)]. Significant interaction with a medium-sized effect for the model was found for the subscales of Exhaustion [*f*^2^ = 0.243, *B* = 0.154, SE = 0.057, *t* = −2.7, CIs = (0.042, 0.266)], whereas small-sized significant effects were found for the model including Cognitive impairment [*f*^2^ = 0.037, *B* = 0.093, SE = 0.047, *t* = −2.01, CIs = (0.002, 0.185)] and Emotional impairment [*f*^2^ = 0.069, *B* = 0.100, SE = 0.046, *t* = −2.15, CIs = (0.009, 0.191)]. All mirrored the conditional effects of the general scale.

## 4 Discussion

### 4.1 Key findings and interpretation

Based on the JD-R theory (8), the study expands its application to migrant workers, demonstrating that social trust functions as a key occupational health resource contributing to a relatively small body of research on this topic (48, 49). The findings suggest that fostering trust-based and linguistically inclusive work environments can increase wellbeing and mitigate burnout—both of which are critical for workplace social inclusion and occupational health. Moreover, the findings extend JD–R theory by demonstrating that the potential of social resources to influence engagement and burnout is conditional upon migrants' linguistic capacity to access and utilize them.

The respondents reached a moderate level of work engagement, similarly to a group of Polish migrant workers in Great Britain studied by Turska and Mochnacka (49) and a multinational migrant group studied by Martinescu et al. (28). Burnout likewise reached a moderate level among respondents.

Hypothesis 1, stating that burnout and work engagement are predicted by coworker and supervisor trust, was fully confirmed for all burnout's and almost all engagement's subscales. This suggests that trust as a resource might be especially valuable for migrant workers in a culturally diverse workplace. As shown by Lahti and Valo (6), in such a context, misunderstandings and tensions can be common. For example, as Aalto et al. (7) has shown, migrant workers feel burdened by lack of professional support more than their native colleagues. Despite this, migrants are often motivated to interact and develop relationships with their native colleagues and supervisors (6, 22, 50). Moreover, especially those who did not want or did not manage to integrate into the host society, experience most of their social life at work (51), which may be another explanation for the value of trust-based workplace relationships to them.

Both kinds of trust among migrants can therefore serve as a resource and positively influence work engagement. However, the facilitating role of coworker trust is conditional upon the host country's language proficiency. Hypothesis 2a stated that such ability would moderate the prediction of work engagement by coworker and supervisor trust and has been confirmed for the first both mentioned kinds of trust. More specifically, moderation was found for its relationship with the general scale of work engagement and the absorption subscale. In each case the relationship was stronger among workers who were proficient in using language. Migrants proficient in the host language may therefore derive greater benefit from social resources due to possible better communication and deeper relational exchanges. Interestingly, no moderation effect was found for supervisor trust. Such a result may be surprising considering that language proficiency has been shown to increase gains from interacting with one's supervisor (36). A possible explanation might be based upon the fact that good language skills have an important function in developing better and deeper relationships at work (6). Although this might be crucial in relationships with coworkers, interactions with one's supervisor, which are often marked by power distance, may have a less personal character and therefore not require good language proficiency. One should also acknowledge alternative explanations to the moderating role of language proficiency. Possible factors underlying the described mechanisms might be the length of stay and cultural distance/proximity, which might impact both language proficiency and workplace relationships. The latter may also be influenced by the degree of acculturation which was not measured in our study.

To our surprise, contrary to the moderating role of the host country's language proficiency, we did not find any significant moderation effect for work language proficiency. Therefore, the Hypothesis 2b was not confirmed. A possible explanation might be that the language predominantly used at work might not always be the default, most comfortable and common language used for internal communication. In such a case, knowing the language mostly used at work does not facilitate the use of social resources to increase work engagement. Future research should explore how one's work language proficiency influences internal communication quality in various aspects, e.g., change communication (52, 53).

Finally, Hypothesis 3, stating that contact frequency with co-national coworkers will moderate the relationship between coworker trust and burnout was confirmed for the general scale of burnout, as well as for its subscales: Exhaustion, Cognitive impairment, and Emotional impairment. The relationship was stronger among those who do not interact with their co-national colleagues when coworker trust was a predictor. Such results may indicate that contact with a minority group at work decreases the significance of social relationships with coworkers. Such a pattern has indeed been identified in in-depth interviews with temporary migrant workers by Lahti and Valo (6), who described such behavior as forming *cultural cliques*. As the scholars found in their study, migrants may provide themselves with the needed comfort, reassurance and self-validation, creating a comfortable environment which does not encourage to undertake the endeavor of integrating with native coworkers, leading to long-term isolation. Our study extends the qualitative findings of Lahti and Valo by showing the direct relationship of co-national coworker contact between workplace trust and burnout, emphasizing its work-health and wellbeing consequence. One might however suggest another possible explanation of the identified phenomenon. Situations when groups of migrants from the same country work together often occur when staffing agencies hire multiple employees at the home country and send them to a foreign company to fulfill a contract limited in time. In such a case, one might not be interested in developing mutual-trust-based relationships at work considering that these would only last for a short period.

The results of our study add to the relatively small group of existing research on the antecedents of migrants' work engagement. One can name, for example, a study by Turska and Mochnacka, who have examined the influence of Berry's acculturation strategies (40) on work engagement. On the other hand, Martinescu et al. (28) focused on objective environmental factors, showing how job skill level predicts (with a moderation of citizenship status) depressive states, which further impact migrant's work engagement. Our present study also extends on our previous work (35) by shifting the focus from finding particular workplace resources to showing how preconditions allow to use them. In this way we emphasize that access to social resources alone may be insufficient for migrant workers if linguistic or social demands limit their use. The current study also differentiates between relationships with coworkers and supervisors and highlights asymmetries between them.

### 4.2 Limitations

Several limitations of the study should be acknowledged. First of all, one should mention that the collected data were based on respondents' self-reports. When assessing one's professional competency and language skills, self-reporting may be a source of bias and, despite the use of validated scales and theoretically distinct constructs, produce common method variance. Another limitation is that all variables were measured at a single point in time, preventing an analysis of dynamic processes, such as the development of work engagement and burnout symptoms or trust building. Future research should respond to the call by Bakker et al. (11–13) to study the dynamics of the work engagement and burnout as a process. Furthermore, the cross-sectional design of the study did not enable one to draw conclusions regarding causality relationships between the studied factors. Although the studied relationships are theoretically grounded in the Job Demands–Resources framework, longitudinal or experimental studies would be needed to confirm the directions of the relationships with more confidence. It should be also noted that data collection relied solely on an online survey, which, despite its advantages, also has certain drawbacks—such as the inability to reach potential participants who are not active online, as well as to ask follow-up questions or control the environment during the study. Further research may be based on a mixed-methods approach to delve deeper into the functioning mechanisms within the social environment of the multicultural workplace. Similarly, using a more complex and objective way of language proficiency measurement would enable its more complex analysis. Finally, considering the number of tested relationships, one cannot fully exclude the possibility of Type I error. However, it should be mentioned that hypotheses had strong theoretical foundations.

Additionally, the research sample had a few constraints, one of which is the small number of participants aged 50 and above. This was however to be expected, as this group is usually underrepresented in working migrant population (1). Previous studies (54) suggest that younger and more educated migrants tend to adapt more quickly to a new country's environment, which may influence the findings. Moreover, the study sample was predominantly female, with twice as many women as men. Research by Milewski and Ruszczak-Żbikowska (55) indicates that migrant women's employment patterns differ from those of men, which may also impact social relationship-building. It must be also mentioned that the results should only be generalized to participants' countries of stay, due to cultural conditions. Although the survey sample included participants from 27 countries, most of them were concentrated in Europe or North America. Particular countries may also differ in cultural distance from the participant group or have different social, governmental, or labor market characteristics. The fact that all participants stemmed from a relatively similar cultural background should also be taken into account. Finally, the participants were mostly highly educated (67.6%) which may have created a bias as education is associated with higher language proficiency (56). Such bias may also be present among particular occupations—future research should collect data on occupation type and industry to control for this factor. Planned length of stay in the host country may also be an important factor in shaping decisions regarding language learning, engaging at work or building workplace relationships. Therefore, it should be considered in future research as well.

Finally, some of the participants were recruited via snowball sampling. This might create a bias when studying variables related to interactions within workplace and national community, as those who have received invitations via this method must have interacted or at least been related to diaspora communities. Such recruitment method leaves very little possibility to reach those who do not keep in touch with their fellow nationals.

### 4.3 Practical implications

From a public health perspective, improving migrants' access to psychosocial resources in the workplace and focusing on reducing psychosocial risks refers to United Nations' SDG 3 (*Good Health and Wellbeing*) and SDG 8 (*Decent Work and Economic Growth*) (2). Employers, policymakers, and occupational health practitioners should focus on interventions and strategies that support the following fields: intercultural communication, language support and help in building trust-based relationships within diverse teams. Importantly, occupational health policies aimed at migrants should reach beyond individual resources. Individual resilience has a strong impact on migrant workers' social environment where other resources and demands may influence occupational health and wellbeing. In consequence, interventions aimed only at individual resources and ignoring the social context of the workplace may be unsuccessful. Therefore, companies and state policymakers should not only put emphasis on language training, but also simultaneously support intercultural team interaction and trust-building initiatives. Policymakers and managers must also recognize the protective role of nation-based social ties. Integration policies may increase the negative impact of distrust on burnout if putting too much pressure on forming relationships with host-country natives at the cost of reducing contact with fellow nationals.

Building on our findings, we would also like to suggest concrete policies. To focus on coworker and supervisor trust and to put emphasis on developing relationships with native coworkers, we suggest that the employer should encourage the employee to integrate into the organization's community right after joining it, during onboarding. Such a process should be based on specific milestones based on gradual exposure to complex tasks but also ensure that the person will be introduced to the workplace community. Such process might be coordinated by a trained trust-person with high cultural competency. The role of this person would be to introduce the newcomer to all team members, present the company and provide answers to questions. To ensure better communication and avoid misunderstandings, onboarding should also include passing the knowledge on explicit organizational norms regarding how feedback is given, how conflicts are to be handled and who to ask for help. Moreover, a good practice is also to pass cultural norms regarding informal communication and building relationships outside of work context—an example of such a process has been described by Ortlieb and Ressi (57). This may help new migrant employees communicate with their colleagues (and even supervisors) in a more diversified, not only formal way, and therefore enhance trust building. It is worth mentioning that also managers might take part in training focused on recognizing linguistic and cultural differences or inclusive communication practices. Emphasis should of course also be put on language learning: for blue collar jobs in which advanced communication is not crucial for effective work, the necessary level of language skills (using the CEFR classification) might usually be level B1. In jobs where language proficiency is required to conduct everyday tasks, level B2 should be the required minimum. As our results show, proficiency in the language used predominantly at work had no significant impact on the relationship between trust and work engagement. Therefore, organizations using, e.g., English as their lingua franca, should put emphasis on the learning of host country's language, regardless of migrant workers' English proficiency.

The completion of particular goals can be supervised using performance indicators (KPIs). Some of them may be tied to the completion of milestones in government-provided integration courses or courses/mentoring programs organized or outsourced by the employer. Besides the individual level of appraisal, other goals and performance indicators may be set on the organizational level, e.g., retention or absenteeism rates of migrant workers or onboarding completion rates.

To provide equal chances for those who are not able to seek opportunities for integration on the internet, government institutions should reach out to migrants, communicating various opportunities and information about the local labor market specifics even before they begin to work (e.g., during visa-obtaining procedures or in refugee camps). Different programs or events should also be available at local job centers, unions and migrant welcoming centers.

To sum up, our suggestions focus on increasing trust within two main paths: encouraging language training and integrating migrant workers into native workplace communities. One should mention that these two have different costs and bring different kinds of results. Structured language training is usually an expensive process (e.g., due to provided materials and paid training time), which needs time-horizons, but offers more measurable results, which further enable employee's access to a broad range of workplace resources. On the other hand, initiatives which aim to integrate the migrant worker into the native community, like mentoring, or facilitating informal peer groups often require lower financial investment and can be implemented quickly but have narrower, short-time effects.

### 4.4 Future research

Future research should investigate how expectations of temporary or long stays abroad influence work relationships. Another point of future interest may be how interaction with migrants' coworkers (fellow nationals and native colleagues) also influences crossover effects, i.e., situations when states experienced by one person affect the level of states of another person. Such effects have been identified for sharing the states of work engagement (58) and work addiction (59) in the general population on multinational samples. Moreover, a useful direction in future research would be to focus on a group with the same or similar language, but different cultural or national background (e.g., Arabic or Latino societies) to diversify the influence of these two factors.

Furthermore, future studies may investigate the dynamic processes forming these relationships and explore particular intervention strategies aiming to prevent burnout and promote engagement in culturally diverse work environments. Use of different methods may allow one to draw conclusions regarding causality of the studied relationships, as well as help to reduce common method variance.

Finally, future research should further explore the labor activity of groups with double disadvantage, i.e., those discriminated not only due to their migrant status but also additionally based on gender, age, and other potential factors which can be seen as handicaps on the labor market.

### 4.5 Summary

The study demonstrates that social and linguistic factors play a crucial role in shaping the occupational health of migrant workers. Trust in coworkers and supervisors serves as a significant resource supporting migrant workers' engagement and buffering against burnout. Therefore, both kinds of trust can be interpreted as resources in the understanding of the JD-R theory (8). Moreover, lack of contact with co-national coworkers enhanced the prediction of burnout by coworker distrust. Host country's language proficiency has on the other hand moderated the relationship between coworker trust and work engagement, being therefore a factor facilitating the utilization of other resources. On the contrary, no such effect has been found for work language proficiency. Future studies should focus on identifying the underlying mechanisms and dynamics of the presented relationships.

## Data Availability

The raw data supporting the conclusions of this article will be made available by the authors, without undue reservation.
