# Correction: Supervisor and coworker trust as predictors of work engagement and burnout among migrant workers: the moderating role of language proficiency and contact with co-national coworkers

**DOI:** 10.3389/fpubh.2026.1839045

**Published:** 2026-05-29

**Authors:** Michał Kulisz, Antoni Wontorczyk

**Affiliations:** 1 Doctoral School in Social Sciences, Jagiellonian University, Kraków, Poland; 2 Faculty of Management and Social Communication, Institute of Applied Psychology, Jagiellonian University, Kraków, Poland

**Keywords:** burnout, JD-R theory, language proficiency, migration, psychosocial resources, work engagement, workplace trust

Text corrections have been made to the following sections:

**Results**, *subsection 3.2 Moderation analysis, first paragraph*

The original sentence read “Unstandardized coefficients (B) are reported to facilitate interpretation of effects, while Cohen's f2 is reported to quantify the incremental effect size of interaction terms.”

The corrected sentence reads “Unstandardized coefficients (B) are reported to facilitate interpretation of effects, while Cohen's f^2^ is reported to quantify the overall effect size of the regression models.”

**Results**, *subsection 3.2 Moderation analysis, 3.2.1 Host country's language proficiency as a Moderator, first paragraph*

The original sentence read “The moderation of the relationship between coworker trust and the general scale of work engagement by host country's language proficiency was found to be significant and of medium size”

The corrected sentence reads “The moderation of the relationship between coworker trust and the general scale of work engagement by host country's language proficiency was found to be significant. The overall model demonstrated a moderate effect size”.

The original sentence read “Significant and relatively low-sized moderation effects have also been found for the Absorption subscale”.

The corrected sentence reads “Significant moderation effects with low-sized model effects size have also been found for the Absorption subscale”.

**Results**, *subsection 3.2 Moderation analysis, 3.2.2 Co-national coworker contact as moderator, first paragraph*

The original sentence read “The relationship between coworker trust and the general burnout scale was significantly moderated by co-national coworker contact and reached a medium-sized effect”.

The corrected sentence reads “The relationship between coworker trust and the general burnout scale was significantly moderated by co-national coworker contact in a model reaching a medium-sized effect”.

The original sentence read “Significant, medium-sized interaction was found for the subscales of Exhaustion [*f*^2^ = 0.243, *B* = 0.154, SE = 0.057, *t* = −2.7, CIs = (0.042, 0.266)], whereas small-sized significant effects were found for Cognitive impairment”.

The corrected sentence reads “Significant interaction with a medium-sized effect for the model was found for the subscales of Exhaustion [*f*^2^ = 0.243, *B* = 0.154, SE = 0.057, *t* = −2.7, CIs = (0.042, 0.266)], whereas small-sized significant effects were found for the model including Cognitive impairment”.

The original version of this article has been updated.

